# Cerebro-Spinal Meningitis in Great Britain

**Published:** 1906-07-21

**Authors:** 


### CEREBRO-SPINAL MENINGITIS IN GREAT BRITAIN.

THIS disease in its typical and epidemic form is somewhat of a rarity in these islands. This typical form is associated with the purpuric eruption which has obtained for the malady the name of "spotted fever." In this shape it appears with considerable frequency in America, and in this shape it visits us sporadically. The organism which causes cerebro-spinal meningitis is a Gram-negative diplococcus, whose distinctive peculiarity lies in the fact that films of the pus formed by its action upon the membranes of the central nervous system show that the organism is mainly intra-cellular. Hence the name diplococcus intra-cellularis meningitidis, which was given to it by Weichselbaum. It is believed that the organism gains access to the meninges by way of the cribriform plate of the ethmoid bone. But there is not much evidence for this assumption beyond the fact that evidences of lesion within the skull are most marked about the base of the brain and the anterior lobes.

We have said that the disease in its typical form is rare in this country; but its occasional occurrence is undoubted. No better evidence of identification can be required than that provided by Dr. F. W. Andrewes [1] in the recently published case of a medical man who died very suddenly of this malady. The story of the case was this: A man of 53 years went to bed one night feeling rather out of sorts. The next morning he felt ill, and did not get up, although he did not think it necessary to ask for medical attendance. By mid-day purpuric spots made their appearance on his face, and his sense of illness became more pronounced. By 5.30 P.M. of the same day he was moribund, and covered from head to foot with an intense purpuric eruption. He died soon after 6 P.M. Films of blood withdrawn about half an hour before death showed that large cocci were present in numbers, and that they were exclusively intracellular, being inclosed in pairs or groups of half-a-dozen within the polynuclear leucocytes. Blood-cultures revealed a pure growth of an organism identical with the diplococcus of Weichselbaum.

This may be taken, therefore, as an example of fulminating cerebro-spinal meningitis; strictly it represented a very virulent septicæmia due to the meningo-coccus, the systemic invasion being so rapid as to anticipate by death the appearance of definite meningeal symptoms. The post-mortem appearances conformed to what might be expected in such an acute septicæmia. Hæmorrhages were extensive both in the intestines and supra-renal bodies, while the brain was the seat of large sub-arachnoid hæmorrhages situated chiefly over the frontal lobes.

Now, although cerebro-spinal meningitis of the type above mentioned is quite uncommon amongst us, there is an analogous disease which frequently victimises members of our infantile population and appears to have a somewhat epidemic prevalence. This is the disease originally described by Gee and Barlow a good many years ago under the title of "cervical opisthotonus of infants," and more commonly known at this time as "posterior basic meningitis." It is a disease affecting in a very large proportion of all cases infants below the age of one year, and very often infants who appear to be in excellent bodily health. In this last particular it imitates acute anterior polio-myelitis, which seems especially prone to attack well-nourished children. The symptoms of onset are somewhat indefinite, but as a rule vomiting is early in evidence, and may occur to an extreme extent. As a result of this persistent vomiting, nutrition very soon begins to fail, and may give place to profound emaciation as the disease progresses. After a few days, as a rule, but occasionally before any other symptoms have manifested themselves, it is observed that the infant when left alone tends to throw his head back. This tendency before long permanently asserts itself, the head being definitely retracted, and all efforts to replace it in a normal position being resisted by a rigidity of the dorsal cervical muscles and accompanied by evidences of pain. But quite often in the absence of disturbance children so affected have little appearance of grave illness. The appetite is not very much affected, and this often when the ingestion of food is promptly followed by vomiting. Before long, as a rule, there appears evidence of an increasing intra-cranial tension, manifested by a progressive hardening of the anterior fontanelle. In a well-marked case the anterior fontanelle may appear as a tense rounded eminence upon the vertex, while all sign of the customary pulsation is banished from it. There is a slight degree of irregular fever. Sight is often impaired, even to complete blindness, but optic neuritis is very uncommon. Ocular paralyses and convulsive seizures are both exceptional. The malady is generally protracted. Wasting progresses, while the loss of flesh and the frequent vomiting combine to produce the retracted or "carinated" shape of the belly which is one characteristic of the established disease. The cervical rigidity may extend to the lower portions of the spine and produce the extremest degrees of general tonic opisthotonus.

After several weeks of such an illness the patient may die of exhaustion, or, not infrequently, of some intercurrent digestive disorder. In such a case autopsy will show a deposit of pus, more or less copious according to the duration of the disease, upon the base of the brain (especially about the posterior portion), and upon the spinal membranes. With this deposit there generally co-exists a variable degree of internal hydrocephalus due to the inflammatory blocking of the foramen of Magendie in the roof of the fourth ventricle. It is this blocking which gives rise to the increasing tension of the anterior fontanelle which marks the disease. The rest of the body shows no lesions characteristic of the malady.

In a favourable case, after some six weeks or so, signs of amelioration in the course of the disease begin to make their appearance. The rigidity of the neck becomes less extreme, and the frequency of the vomiting diminishes. As a consequence a better state of nutrition is established and an apparently complete cure ultimately results. The qualification "apparently" is necessary because it is impossible to be assured at the time that no permanent intellectual damage has resulted. In many cases the basal inflammation and its consequences result in a permanent condition of hydrocephalus, but not by any means always. The blindness which often accompanies the active stage of the disease may appear to be complete and yet disappear entirely.

This infantile disorder presents, as we have said, certain well-marked analogies to epidemic cerebro-spinal fever. It is cerebro-spinal in its immediate lesions, and it exhibits a certain, but very limited, epidemicity. Further, bacteriological considerations indicate a close similarity, amounting, in the opinion of many observers, to an actual identity, of the causal organism in the two diseases. If the spinal theca be punctured in the lumbar region, after the fashion introduced by Quincke, the fluid withdrawn is found in a large proportion of cases to contain an intra-cellular diplococcus indistinguishable from that of Weichselbaum. At the same time the curious and general limitation of the disease to the first twelve months of life, combined with certain minor clinical differences, such as the absence of petechiæ, indicates a definite, though possibly slight, variation in the characteristics of the causal element.

As regards the diagnosis there is seldom great difficulty if the case be typical. Occasionally it may be hard to say early in its course whether the lesion is tuberculous or not. The points of distinction upon which reliance is to be placed are these: That in the first year of life an apparently primary meningitis is more likely to be cerebro-spinal than tuberculous; that retraction of the head and bulging of the fontanelle are much more marked in the cerebro-spinal variety. That cerebral symptoms such as irritability, fretfulness, drowsiness, and photophobia are particularly associated with the tuberculous variety. That convulsions, ocular palsies and optic neuritis are quite common in the course of tuberculous meningitis, but rare in the cerebro-spinal disease. An absolute diagnosis can only be made at an early stage by means of lumbar puncture. It is of high importance in this connection to recognise that retraction of the head, *per se*, is a most unreliable index of meningeal trouble in early infancy. Although the circumstance lacks explanation it is an undoubted fact that head-retraction, even to a well-marked extent, may accompany a variety of minor disturbances, gastro-intestinal and other. We have seen an infant thus affected, and one of whom it was credibly asserted that the retraction had persisted for three weeks, yet recover completely in three days, a sequence which negatives absolutely any theory of meningitis.

There is no specific remedy for the disease. Occasionally good results seem to attend the use of potassium iodide in large doses. Nor do large doses of this drug appear to exert any evil effect. In one case within our knowledge an infant of less than one year was given ten grains of this iodide thrice daily for a long period without any symptoms of iodism. But it is well to increase the dose gradually. Lumbar puncture for the withdrawal of the cerebrospinal fluid has been advocated as a therapeutic measure, but it is of little value. A few attempts have been made to obtain a specific serum for the individual organism by the immunisation of animals with cultures grown from the spinal fluid, but no definite information is available as to the value of the method. The treatment must in the main be general and devoted to the maintenance of nutrition and the avoidance of bed-sores.

